# Equine mesenchymal stromal cells dual-primed with TGF-β1 and IL-1β or TNF-α improve epithelial healing

**DOI:** 10.21203/rs.3.rs-10072744/v1

**Published:** 2026-06-22

**Authors:** Liliana C. Patiño, Hammed Ayansola, Younggeon Jin, Alix K. Berglund

**Affiliations:** University of Maryland, College Park; University of Maryland, College Park; University of Maryland, College Park; University of Maryland, College Park

**Keywords:** equine, mesenchymal stromal cell, TGF-β, colonoid, wound healing

## Abstract

**Background:**

Mesenchymal stromal cells (MSCs) secrete paracrine factors that contribute to their ability to promote tissue healing and regeneration. Priming equine MSCs with cytokines like TGF-β is known to enhance expression of growth factors and extracellular matrix molecules, which may increase their potential for treating certain injuries including epithelial wounds. We hypothesized that dual priming equine MSCs with TGF-β1 plus the inflammatory cytokines IL-1β or TNF-α would further increase expression of paracrine factors relevant to epithelial healing and their therapeutic potential.

**Results:**

Priming equine MSCs did not affect the morphology or viability of the cells. Equine MSCs dual-primed with TGF-β1 + IL-1β or TGF-β1 + TNF-α had increased expression of PGE2, AREG, and HBEGF. Conditioned media from both naïve and primed MSCs increased wound closure rates and dual-primed MSCs significantly increased the growth of colonoids in a co-culture model. Priming human induced MSCs did not stimulate the same changes in paracrine factor expression seen in equine MSCs.

**Conclusions:**

Priming equine MSCs with TGF-β1 + IL-1β or TGF-β1 + TNF-α is a promising strategy for generating cells or conditioned media with the necessary growth factors and cytokines for promoting epithelial healing.

## 1. INTRODUCTION

Mesenchymal stromal cells (MSCs) are of interest as a regenerative therapy in veterinary and human medicine due to their ability to modulate inflammatory responses and promote endogenous tissue healing [1]. A major contributor to the regenerative capabilities of MSCs are the bioactive factors they produce; MSCs secrete anti-inflammatory cytokines that inhibit the immune system from further damaging tissues after injury, growth factors that stimulate cellular proliferation and progenitor cell differentiation, and extracellular matrix molecules that directly contribute to tissue remodeling and repair [2, 3]. Due to the secretion of these paracrine factors, MSC-based therapies have considerable potential for treating epithelial injuries, particularly complex and non-healing chronic wounds and inflammatory bowel diseases.

Despite MSCs showing remarkable potential in *in vitro* assays and preclinical rodent models, results in clinical trials have been inconsistent. A lack of standardized culture methods, variability in the quality of donor cells, issues with immunocompatibility, and unidentified critical quality attributes all contribute to the lack of success in clinical trials [4, 5]. The use of MSC conditioned media, which is acellular, may overcome the issue of immunocompatibility and conditioned media can be easily screened prior to use for functional quality [6–8]. Conditioned media can also be lyophilized to create an off-the-shelf product that can potentially be stored at room temperature or normal refrigerated conditions. Another approach to standardizing culture methods and reducing variation in MSC product quality is to “prime” MSCs *in vitro* using a specific culture system with the goal of enhancing the production of bioactive factors [9]. One priming strategy is to stimulate MSCs with pro-inflammatory cytokines like IFN-g, IL-1β, or TNF-α as this increases the production of anti-inflammatory cytokines from the MSCs [10]. We have previously shown that priming MSCs *in vitro* with TGF-β2 increases the production of growth factors and extracellular matrix molecules important for promoting tendon repair [11]. Treating MSCs with TGF-β therefore shows potential for improving the regenerative capabilities of the cells, but dual priming strategies with TGF-β and an inflammatory cytokine may further improve their therapeutic functions [12].

In this study, we explored whether equine MSCs primed with TGF-β1 alone or TGF-β1 in combination with IL-1β or TNF-α were superior at promoting epithelial cell healing compared to naïve MSCs. Human induced MSCs (iMSCs) derived from induced pluripotent stem cells (iPSCs) were also used to compare the results of priming between equine and human cells. We found that TGF-β1 + IL-1β dual-primed MSCs had increased expression of prostaglandin E2 (PGE2), increased gene expression of growth factors, increased the migration of epithelial cells, increased colonoid proliferation in MSCs compared to naïve MSCs. Additionally, upregulation of genes associated with immunomodulation and repair was much stronger in primed equine MSCs compared to primed human iMSCs.

## 2. METHODS

### 2.1 Animal use and welfare

Six systemically healthy horses were utilized in this study for sternal bone marrow harvest as approved under protocol #20–454 for post-mortem collection by the Institutional Animal Care and Use Committee of North Carolina State University. The horses were between 5 and 16 years of age and of mixed breeds. An equal number of mares and geldings were used in the study.

### 2.2 MSC isolation and culture

Banked equine bone marrow-derived MSCs were utilized for this study and isolated as previously described [11]. MSCs were thawed and cultured in complete media contained 1 g/dl glucose DMEM (Corning), 10% fetal bovine serum (Cytivia), 2 mM L-glutamine, 100 U/ml penicillin and streptomycin, and 1 ng/ml recombinant human basic fibroblast growth factor (bFGF). When cultures reached 80% confluency, MSCs were passaged with Accutase cell-dissociation solution and plated at a density of 6,250 cells per cm^2^ on 100 mm tissue culture plates. MSCs were used in this study at passage 4 or 5. Cell count and viability at each passage were determined using a Cellometer^®^ Auto 2000 and ViaStain^™^ AOPI Staining Solution (Nexcelom Bioscience LLC). MSCs were primed by adding 1 ng/ml recombinant human TGF-β1 and 2 ng/ml IL-1β or 10 ng/ml TNF-α (BioLegend) to the media for 72 hours prior to analysis or co-culture.

For collection of conditioned media, the MSCs were primed as described above for 72 hours then washed twice with PBS and standard media without FBS or bFGF was added to the cultures. The conditioned media was then collected 24 hours later and frozen at −80°C.

### 2.3 Migration assay

Caco-2 cells were obtained from ATCC and cultured at 37°C in 5% CO_2_ in media consisting of DMEM/F12 with 10% FBS (Cytivia), 2 mM L-glutamine, and 1% penicillin/streptomycin. Caco-2 cells were seeded at a density of 1 × 10^4^ cells/cm^2^ and fed every 48 hours with pre-warmed media. When cultures reached 80% confluency, they were passaged with Trypsin-EDTA (0.25%). Caco-2 cells were cultured for 2–3 passages prior to use.

A standard migration assay was performed using silicone cell culture inserts (Ibidi) to create cell free gaps or “wounds.” 3-well culture inserts were inserted into the wells of a 12-well plate. 4 × 10^4^ Caco-2 cells in 70 μl of media were added to each well of the culture inserts and allowed to adhere to the plate overnight. The cell culture inserts were then removed and the cells washed twice in PBS before adding Caco-2 culture media containing 0.5% FBS with or without MSC conditioned media at 30% final volume. Images of each wound were captured every 24 hours beginning immediately after removing the insert (0 hour) with a digital microscope camera (AmScope) mounted on an Olympus CKX53 inverted microscope. Wounds were manually traced using ImageJ software and the percentage of wound healing was calculated relative to hour 0 using the formula [(Area at t = 0 hr – Area at t = 24 hr)/Area at t = 0 hr]*100.

### 2.4 2D Human Colonoid Expansion Culture System

For the co-culture study, frozen colonoids from 14 human donors were obtained from Dr. Scott Magness’s lab and expanded into 2D epithelial monolayers on hydrogel-coated plates, following a standard protocol [13]. The monolayers were maintained in an enriched expansion media consisting of 50% L-WRN conditioned medium and 50% Advanced DMEM/F12 (Gibco). The base medium was supplemented with 1X GlutaMAX, 10 mM HEPES, 1X B27, 1X antibiotic-antimycotic, 1.25 mM N-acetyl cysteine, 50 ng/ml human EGF, 10 mM nicotinamide, 10 mM gastrin, 0.1 μM PGE2, 3 μM SB202190, and 10 μM Y-27632. The media was replaced every other day. Upon reaching 80% confluence, the monolayers were passaged using collagenase IV.

### 2.5 Matrigel embedded 3D colonoid co-culture system

For the 3D co-culture experiment, colonoids from passage 7 were used to investigate the effects of naïve and primed MSCs on colonoid growth. Briefly, the epithelial fragments about 35 μM in size were isolated from the subcultured 2D monolayers using a p200 pipette tip. These fragments were resuspended in ice-cold Matrigel and embedded with naïve or primed MSCs from a single equine donor. 10 μl of ice-cold Matrigel containing either 15 fragments only or directly co-embedded with 5,000 MSCs per well as technical replicates, with each treatment group having 2 replicates. The Matrigel was allowed to polymerize at 37C for 15 minutes. After polymerization, 3D culture media was added to the Matrigel dome containing 50% L-WRN conditioned medium, 50% advanced DMEM/F12, 10 mM HEPES, 50 ng/mL EGF, 1 mM N-acetylcysteine, 50 μg/mL Primocin, 500 nM A-8301, and 10 μM Y-27632. The media was replaced every other day until day 10. Timepoint imaging of the colonoids was performed to assess morphology. The area of five colonoids per well was measured using the freehand tool in ImageJ and then the mean for each duplicate was calculated relative to the control (colonoids alone).

### 2.5 Human iMSCs induction.

Monoclonal iPSCs derived from dermal fibroblasts and reprogrammed using mRNA were obtained from the New York Stem Cell Foundation (cell line 051061–01-MMR). iPSCs grown as aggregates (passage #3) in StemFlex medium (Gibco) were induced to iMSCs using a modified direct switch method [14]. Briefly, three days after passaging, iPSCs were switched from StemFlex to MSC media consisting of low-glucose DMEM, 10% fetal bovine serum (Cytivia), 2 mM L-glutamine, 100 U/ml penicillin and streptomycin, and 3 ng/ml recombinant human bFGF for 14 days. P1 cells were passaged and seeded at 40,000 cells per cm^2^ with subsequent passages seeded at 8,000 cells per cm^2^. At passage 3, MSC surface markers were detected using flow cytometry. iMSCs were positive for CD90, CD73 and CD105 and negative for CD24, CD45 and CD11b (**Supplementary Fig. 1**). As with the equine MSCs, passage 5 iMSCs were primed by treating the cells for 72 hours with 1 ng/ml recombinant human TGF-β1 and 2 ng/ml IL-1β or 10 ng/ml TNF-α (BioLegend).

### 2.6 Flow cytometry

To measure MHC I surface expression, MSCs were washed in PBS and incubated with 10% goat serum prior to labeling with primary antibody. Equine MSCs were labeled with mouse anti-horse MHC I or MHC II antibody (clones cz3 and cz11 respectively, gift from Dr. Doug Antczak) followed by APC-conjugated goat anti-mouse Ig antibody (BD Biosciences). For iMSC phenotyping, cells were labeled with anti-human CD90 (clone 5E10, BD Biosciences), anti-human CD73 (clone AD2, BD Biosciences), anti-human CD105 (clone SN6h, BD Biosciences), anti-human CD24 (clone SN3, Invitrogen), anti-human CD45 (clone HI30, BD Biosciences) and anti-mouse/human CD11b (clone M1/70, BioLegend). 4’,6’-diamidino-2-phenylindole (DAPI) was added at 500 ng/ml 15 minutes prior to analysis to identify dead cells. Fluorescence of live cells was measured using a BD FACS Celesta flow cytometer equipped with FACSDiva analysis software (BD Biosciences). Data was analyzed using FlowJo v.10 (FlowJo, LLC).

### 2.7 ELISAs

Conditioned media from each horse and treatment group was frozen and stored at −80°C. ELISAs for human PGE2 (Enzo Life Sciences), human TGF-β1 (Quantikine, R&D Systems), and equine LIF (MyBioSource) were performed per manufacturers’ instructions. For PGE2 analysis, conditioned media was diluted 1:100 in reagent diluent.

### 2.8 Real-time quantitative PCR

RNA was extracted from MSCs and iMSCs at the end of the 72-hour priming period. Total RNA was isolated using the RNeasy Mini Kit (Qiagen) and cDNA was synthesized using the iScript cDNA Synthesis kit (Bio-Rad). Transcripts were quantified by real-time PCR analysis using the SsoAdvanced Universal SYBR Green (Bio-Rad) and the CFX Opus 384 system (Bio-Rad). mRNA expression in MSCs and iMSCs was normalized to *HPRT1* and fold change was calculated as the relative gene expression (2^− ΔΔCt^). Primers were generated using Primer-Blast unless otherwise specified with sequences and references listed in Table 1. For all reactions, each condition was performed in triplicate.

### 2.9 Statistical analysis

Statistical analysis was conducted using JMP Pro 15 (SAS Institute Inc). Data were normalized by log transformation and analyzed using a one-way ANOVA followed by a Tukey’s test for multiple comparisons.

## 3. RESULTS

### 3.1 TGF-β priming decreases the MSC population doubling time without affecting morphology.

**Figure 1:**
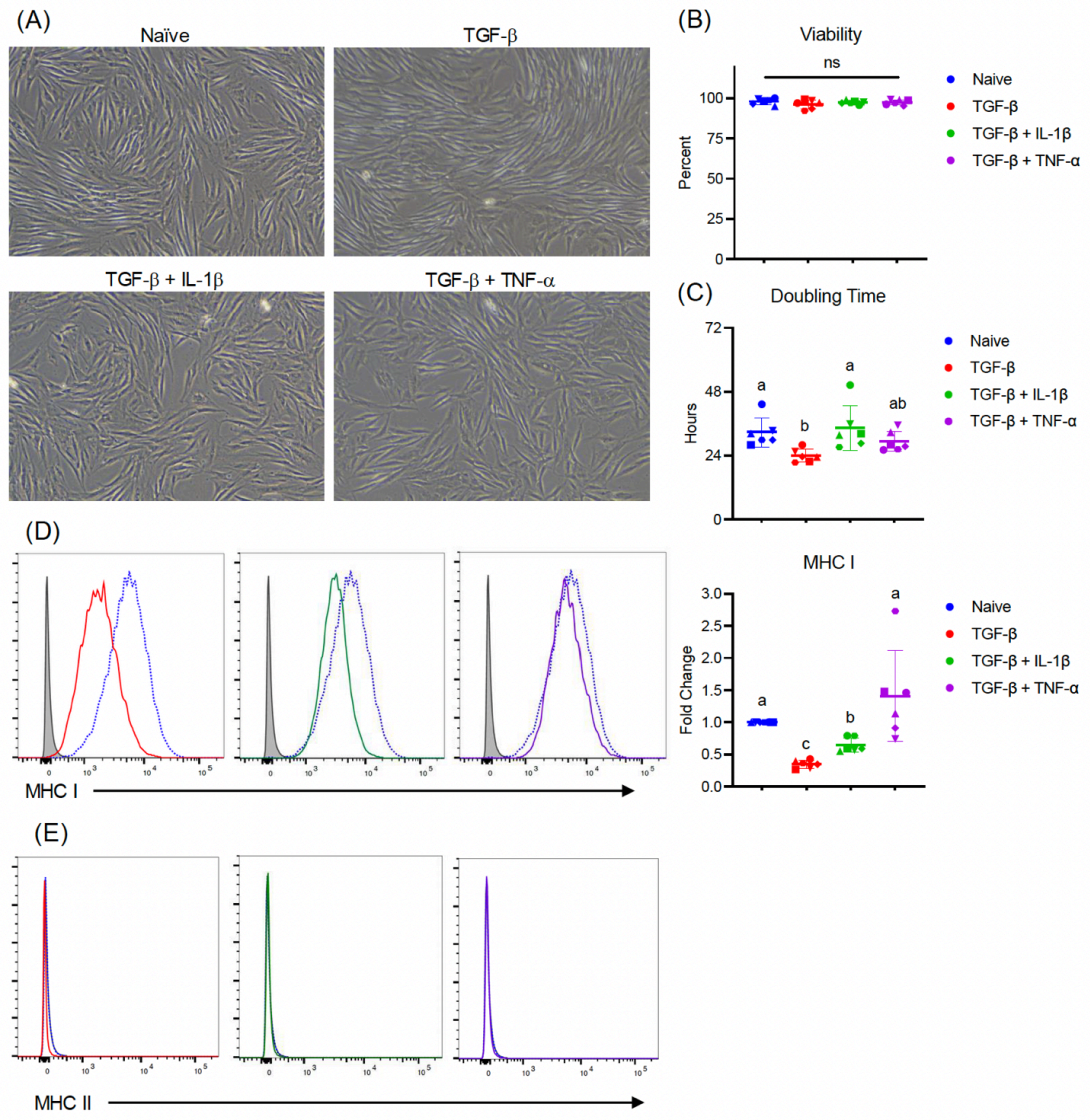
Primed equine MSCs have normal morphology and viability. **A.** Morphology of naïve and primed MSCs. **B.** Viability of naïve and primed MSCs. **C.**Population doubling time of naïve and primed MSCs. **D.** Representative histograms of MHC I expression on primed MSCs compared to naïve MSCs and relative MHC I expression on primed MSCs compared to naïve MSCs. **E.**Representative histograms of MHC II expression on primed MSCs compared to naïve MSCs. Data shown are mean +/− SD for n = 6. Superscript letters indicate statistically significant differences between groups at p < 0.05 as determined by a one-way ANOVA followed by a Tukey’s post-hoc test.

The general characteristics of equine MSCs primed with 1 ng/ml TGF-β1, 1 ng/ml TGF-β1 + 2 ng/ml IL-1β, or 1 ng/ml TGF-β1 + 10 ng/ml TNF-α were first compared to naïve MSCs. There were no significant visual differences in the morphology of the cells following priming nor in the viability of the cells after 72 hours of treatment (Figs. 1A and 1B). Priming with TGF-β1 significantly reduced the population doubling time of the cells compared to naive, but co-priming with IL-1β, and to a lesser extent TNF-α, largely negated this effect (Fig. 1C). In agreement with our previous studies, TGF-β significantly reduced the surface expression of MHC I and the addition of IL-1β slightly dampened this effect (Fig. 1D) [18, 19]. Co-priming MSCs with TNF-α blocked the ability of TGF-β to downregulate MHC I and in some horses increased MHC I surface expression. MHC II expression was not induced with any treatment group (Fig. 1E).

### 3.2 Primed MSCs have altered expression of cytokines and growth factors.

**Figure 2:**
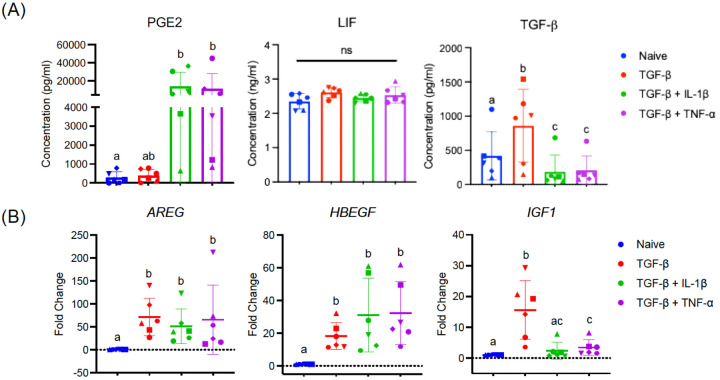
Priming equine MSCs increases expression of epithelial growth factors. **A.** Concentrations of PGE2, LIF, and TGF-b1 in the conditioned media of naïve and primed equine MSCs. **B.** Fold changes in AREG, HBEGF, and IGF1 gene expression in primed equine MSCs relative to naïve MSCs. Data shown are mean +/− SD for n = 6. Superscript letters indicate statistically significant differences between groups at p < 0.05 as determined by a one-way ANOVA followed by a Tukey’s post-hoc test.

To understand how priming the MSCs with TGF-β1 in conjunction with the inflammatory cytokines IL-1β and TNF-α changed the production of key cytokines and growth factors, we measured PGE2, LIF, and TGF-β1 in conditioned media after 72 hours of priming. TGF-β-primed MSCs did not have significantly different PGE2 expression than naïve MSCs, but co-priming with IL-1β or TNF-α significantly increased PGE2 production (Fig. 2A). Expression of LIF was not significantly different between any treatment group, but priming MSCs with TGF-β1 increased TGF-β1 expression while co-priming with IL-1β or TNF-α suppressed expression compared to naïve MSCs. The concentrations of each cytokine were highly variable between individual horses and the exact concentrations measured from each horse in each treatment group are shown in **Supplementary Tables 1–3**. MSCs secrete numerous growth factors that promote epithelial proliferation and migration including amphiregulin (AREG), heparin binding EGF-like growth factor (HBEGF), and insulin-like growth factor-1 (IGF-1). Using qPCR, we found that gene expression of AREG and HBEGF was significantly increased in all primed MSCs compared to naïve (Fig. 2B). Priming with TGF-β alone increased gene expression of IGF1 and co-priming MSCs with IL-1β or TNF-α mostly blocked this effect. As with cytokine expression, gene expression was highly variable between horses. Priming MSCs with TGF-β alone and co-priming with IL-1β or TNF-α therefore has different effects on the secretion of important cytokines and growth factors including PGE2, TGF-β1, and IGF-1.

### 3.3 Conditioned media from naïve and TGF-β + IL-1β MSC improves epithelial cell migration

**Figure 3:**
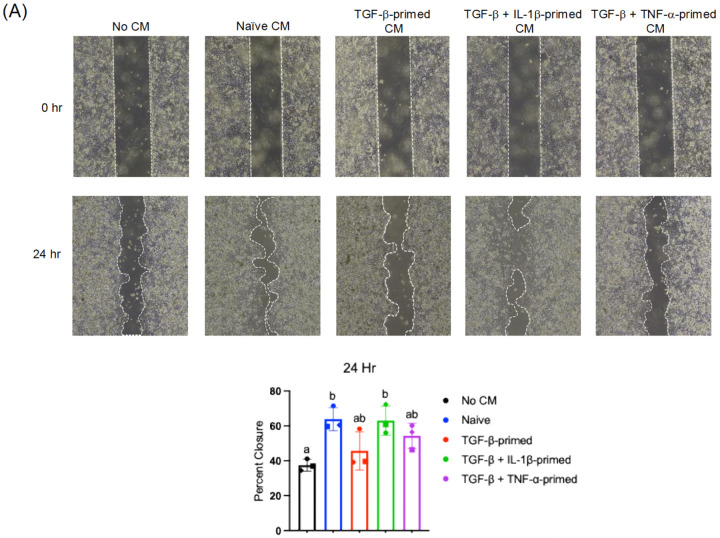
Equine MSC conditioned media promotes wound closure. **A.**Representative images of untreated and MSC conditioned media-treated wounds at 0 and 24 hours. **B.** Percent closure of wounds treated with MSC conditioned media relative to untreated wounds. Data shown are mean +/− SD for n = 3. Superscript letters indicate statistically significant differences between groups at p < 0.05 as determined by a one-way ANOVA followed by a Tukey’s post-hoc test.

To understand how secreted factors from primed MSCs may affect epithelial healing, we added conditioned media from each treatment group to “wounds” made in Caco-2 monolayer cultures and percent closure of the wound was calculated after 24 hours. Conditioned media from all MSC groups improved closure of the wounds compared to wounds not treated with conditioned media (Fig. 3A). Wounds treated with conditioned media from naïve MSCs and TGF-β + IL-1β-primed MSCs had the highest percent closure at 24 hours indicating that secreted factors from both naïve and primed MSCs can improve epithelial migration during wound healing.

### 3.4 TGF-β + IL-1β-primed MSCs promote colonoid proliferation

**Figure 4:**
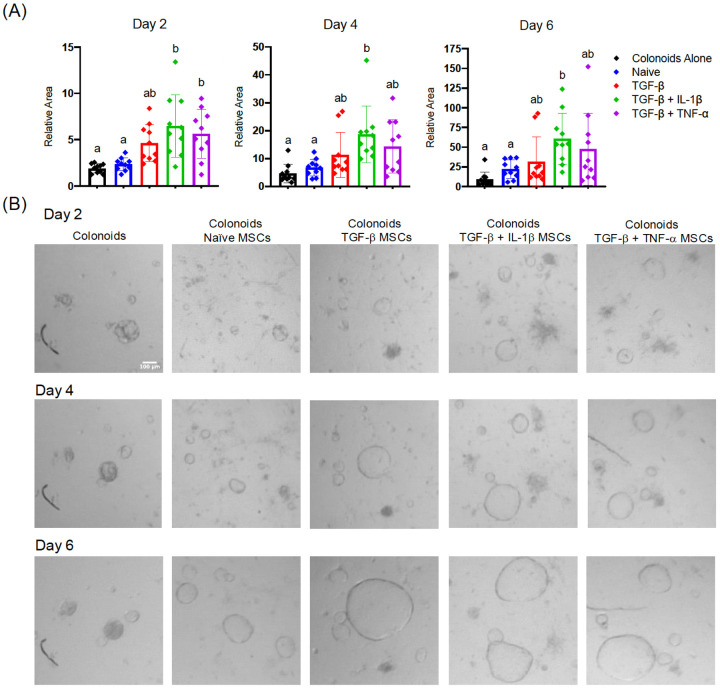
Conditioned media from dual-primed MSCs enhances colonoid growth. **A.**Area of colonoids alone or co-cultured with equine MSCs on day 2, day 4, and day 6 relative to day 0. Data shown are mean +/− SD for n = 10. Superscript letters indicate statistically significant differences between groups at p < 0.05 as determined by a one-way ANOVA followed by a Tukey’s post-hoc test. **B.**Representative images of colonoids from each treatment group at day 2, day 4, and day 6.

Caco-2 cells have historically been used as a model of intestinal epithelium, but 3D colonoid models more accurately reflect intestinal physiology [20]. Human colonoids were suspended in Matrigel with MSCs from each treatment group. Images were taken at 0, 2, 4, and 6 days and the area for 10 colonoids per treatment group was calculated relative to day 0. At no time point was there a significant difference in relative area between colonoids cultured alone and colonoids cultured with naïve MSCs or TGF-β1-primed MSCs, although very large individual colonoids were imaged in the wells with TGF-β1-primed MSCs (Fig. 4A). Colonoids co-cultured with TGF-β1 + IL-1β-primed MSCs had significantly higher relative area at all days compared to colonoids cultured alone or with naïve MSCs. Colonoids co-cultured with TGF-β1 + TNF-α-primed MSCs only had significantly higher relative area at day 2 compared to colonoids cultured alone or with naïve MSCs. This further supports that dual priming MSCs with TGF-β1 + IL-1β or TGF-β1 + TNF-α enhances their ability to promote proliferation of intestinal epithelial cells.

### 3.5 TGF-β + IL-1β- and TGF-β + TNF-α- primed iMSCs promote *HBEGF* expression

**Figure 5:**
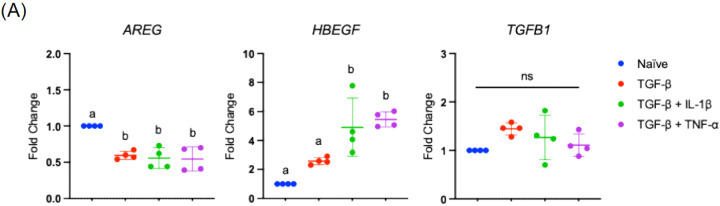
Dual-primed human iMSCs have increased HBEGF expression. **A.**Fold changes in AREG, HBEGF, and TGFB1 gene expression in primed human iMSCs relative to naïve iMSCs. Data shown are mean +/− SD for n = 3. Superscript letters indicate statistically significant differences between groups at p < 0.05 as determined by a one-way ANOVA followed by a Tukey’s post-hoc test.

iMSCs, which are MSC-like cells differentiated from iPSCs, are a potential new regenerative therapy that are reported to have similar immunomodulatory and regenerative potential as conventional MSCs as well as improved scalability [21]. To evaluate if the priming strategy used in equine MSCs also improves the regenerative potential of human iMSCs, we primed iMSCs with TGF-β1 alone and in combination with IL-1β or TNF-α and evaluated the expression of genes *AREG*, *HBEGF*, *TGFB1*, *IGF1* and *PTGES*. Unlike the primed equine MSCs, *AREG* expression was downregulated for the three primed conditions in comparison with naïve iMSCs (Fig. 5A). Notably, *HBEGF* was upregulated in TGF-β1 + IL-1β- and TGF-β1 + TNF-α-primed iMSCs, but to a much lower degree than in equine MSCs. *TGFB1* tended to increase for all the primed conditions, but not significatively. *IGF1* and *PTGES* gene expression was lower than the limit of detection in these cells (data not shown). This demonstrates that there are significant differences in both the constitutive and primed expression of paracrine factors between equine MSCs and human iMSCs.

## 4. DISCUSSION

The purpose of this study was to determine how priming equine MSCs with a combination of TGF-β1 and IL-1β or TNF-α alters the expression of paracrine factors and the functional properties of the cells relevant to epithelial healing. We hypothesized that MSCs primed with both TGF-β1 and an inflammatory cytokine would have increased immunomodulatory and regenerative potential compared to naïve MSCs or MSCs primed with TGF-β1 alone. We demonstrated that equine MSCs primed with TGF-β1 + IL-1β or TGF-β1 + TNF-α have increased expression of cytokines including PGE2 and HBEGF, promote epithelial cell migration, and promote colonoid proliferation. Additionally, we found that the benefits of priming on cytokine expression did not extend to human iMSCs.

We previously reported that priming equine MSCs with TGF-β2 is an efficient strategy to increase the expression of growth factors, such as IGF-1, connective tissue growth factor, and acidic fibroblast growth factor (FGF1), as well as extracellular matrix molecules, including type I collagen and tenascin-C [11]. In a follow-up study, equine bone marrow-derived MSCs dual-primed with TGF-β2 + IL-1β also promoted tenocyte regeneration by increasing tenocyte migration and metabolism and reduced expression of IL-1β-induced extracellular matrix remodeling genes in tenocytes [12]. The results from our current study support that MSCs primed with TGF-β1 + IL-1β promote epithelial healing better than naïve MSCs or when primed with TGF-β1 alone. MSCs primed with TGF-β + TNF-α also had increased expression of relevant paracrine factors, but tended to be less effective at promoting epithelial cell migration and proliferation and had increased MHC I surface expression compared to those primed with IL-1β.

Although MSCs can affect other cells through cell-to-cell contact, the secretion of paracrine factors is the primary method by which they exert their regenerative functions [22]. PGE2 is a major mediated is known to be important for immunomodulation and regeneration in both equine and human MSC therapy [23–27]. PGE2 was detected in the conditioned media of equine MSCs from all treatment groups, but concentrations were significantly higher in conditioned media from the dual-primed MSCs compared to naïve or TGF-β1-primed. PGE2 accelerates cutaneous wound healing, increases regeneration of new hair follicles and sebaceous glands, and reduces scar formation by inhibiting myofibroblast infiltration in mice [28]. The PGE2 signaling pathway also promotes the proliferation of Lgr5 + intestinal stem cells [29, 30]. Consistent with this, exogenous PGE2 has been shown to induce significantly higher levels of cell proliferation in mouse colonoids after 5 days of treatment, along with upregulation of stem cell–related and Wnt signaling–associated gene expression [29]. Increased proliferation of colonic stem cells may explain why colonoid growth was increased when co-cultured with the dual-primed MSCs compared to naïve or TGF-β-primed MSCs. Although conditioned media from naïve and dual-primed MSCs increased migration of Caco-2 cells in wound healing, wound closure rates were not significantly different between naïve and dual-primed MSCs indicating that PGE2 may not be the primary mediator for promoting cell migration in this context.

Growth factors like AREG, HB-EGF, and IGF-1 induce proliferation of multiple cell types involved in wound healing [31, 32]. Upon secretion, these growth factors act through autocrine, paracrine, or endocrine mechanisms by binding membrane or cytoplasmic receptors, thereby activating intracellular signaling cascades that drive tissue repair [33]. AREG is constitutively expressed by diverse cell types of epithelial, mesenchymal, and hematopoietic origin and its expression is upregulated in wounded keratinocytes [34]. How AREG contributes to wound healing is poorly understood, but AREG produced by intestinal myofibroblasts has been shown to stimulate intestinal epithelial proliferation and is essential for PGE2-mediated intestinal epithelial regeneration [35]. As AREG gene expression was increased in all primed treatment groups, together with PGE2 it may have contributed to the increased growth seen in colonoids co-cultured with primed MSCs. HB-EGF is critical for skin healing as wound closure is significantly delayed in HB-EGF knockout mice due to impaired migration of keratinocytes [36]. In rat models, exogenous HB-EGF treatment promotes intestinal restitution, helps restore gut barrier permeability, and protects proliferating intestinal stem cells after injury [37, 38]. Similarly, IGF-1 also promotes migration of epithelial cells and keratinocytes after injury as well as angiogenesis [39, 40]. TGF-β1 expressed by MSCs is important for inhibiting adaptive and innate immune responses and generating Treg cells, but excessive production of TGF-β can also promote fibrosis in wound healing [26, 41–43]. TGF-β1 was only upregulated in TGF-β1-primed MSCs and downregulated in dual-primed MSCs, so dual-primed cells may have a greater benefit in clinical situations where more tissue regeneration than immunosuppression is needed.

In this study, we also compared the effects of priming on equine MSCs and human iMSCs. iMSCs, which can be differentiated from iPSCs using multiple different methods [44] are of increasing interest in human regenerative medicine due to their MSC-like regenerative properties, reduced variability, and scalability [45]. iMSCs are reported to have similar to better immunomodulatory and regenerative properties compared to conventional MSCs [46–49], but there is still much unknown about the secretome of these cells and how the source iPSC cell line and differentiation methods affect their functional properties [50]. There do appear to be significant differences between iMSCs and conventional MSCs including the expression of PGE2. We did not detect significant PGE2 transcript expression in the iMSCs used in this study, which is consistent with findings in earlier studies using iMSCs derived using a TGF-β pathway inhibition method [51]. No priming strategy in this study resulted in expression of PGE2 in iMSCs, but similar to equine MSCs we did detect increased expression of HBEGF after dual priming. More studies are needed to understand the mechanisms of action of iMSCs and how to culture and enhance them to promote epithelial healing.

Our study supports that dual priming equine MSCs with TGF-β1 + IL-1β or TGF-β1 + TNF-α increases expression of critical growth factors for both cutaneous and intestinal epithelial healing. These primed cells or their conditioned media may be an improvement over naïve MSCs or other conventional therapies for treating skin or intestinal wounds in horses. Importantly, we did not see these same effects when human iMSCs were primed. As MSC conditioned media is acellular, equine MSC conditioned media may be a potential therapeutic in human medicine as well, although more studies are needed to determine if MHC I molecules shed into the media are immunogenic. Understanding the composition of equine MSC conditioned media and how its components promote epithelial healing may also help to determine how to human iMSC conditioned media could be improved for clinical use.

## Supplementary Material

Supplementary Files

This is a list of supplementary files associated with this preprint. Click to download.

- SupplementalTable1.docx

## Figures and Tables

**Table 1:** qPCR primer sequences

*Horse*				
Gene		Sequence (5′−3′)	Product Length	Reference
*AREG*	Forward	TGTCGCTCTTGATCCTCGG	116 bp	
	Reverse	CACCTCAAACCCATCAGCACT		
*HBEGF*	Forward	AGAGAGACCCATGTCTTCGGA	154 bp	
	Reverse	TATAGGCGATTTTCCACCGGG		
*HPRT1*	Forward	AATTATGGACAGGACTGAACGG	121 bp	Beekman 2011[15]
	Reverse	ATAATCCAGCAGCTCAGCAAAG		
*IGF1*	Forward	ACGCTCTTCAGTTCGTGTGT	137 bp	
	Reverse	CAGCCTCCTCAGATCACAGC		
*Human*				
Gene		Sequence (5′−3′)	Product Length	Reference
AREG	Forward	GAGCCGACTATGACTACTCAGA	121 bp	PrimerBank [16]
	Reverse	TCACTTTCCGTCTTGTTTTGGG		
HBEGF	Forward	ATCGTGGGGCTTCTCATGTTT	86 bp	PrimerBank
	Reverse	TTAGTCATGCCCAACTTCACTTT		
HPRT1	Forward	GTAATGACCAGTCAACAGGGGAC	177 bp	Lam 2015 [17]
	Reverse	CCAGCAAGCTTGCGACCTTGACCA		
IGF1	Forward	GCTCTTCAGTCGTGTGTGGA	133 bp	PrimerBank
	Reverse	GCCTCCTTAGATCACAGCTCC		
PTGES	Forward	TCCTAACCCTTTTGTCGCCTG	166 bp	PrimerBank
	Reverse	CGCTTCCCAGAGGATCTGC		
TGFB1	Forward	CAATTCCTGGCGATACCTCAG	86 bp	PrimerBank
	Reverse	GCACAACTCCGGTGACATCAA		

## Data Availability

All data is available upon reasonable request.
